# Sinusitis and oroantral fistula in patients with bisphosphonate-associated necrosis of the maxilla

**DOI:** 10.1186/s13005-015-0099-0

**Published:** 2016-01-06

**Authors:** Pit Jacob Voss, Gustavo Vargas Soto, Rainer Schmelzeisen, Kiwako Izumi, Andres Stricker, Gido Bittermann, Philipp Poxleitner

**Affiliations:** Department of Oral and Maxillofacial Surgery, Regional Plastic Surgery, Medical Center - University of Freiburg , Hugstetter Str. 55, 79106 Freiburg im Breisgau, Germany; Department of Oral and Maxillofacial Surgery, Hospital San Juan de Dios, Universidad Latina, San José, Costa Rica; Department of Oral and Maxillofacial Surgery, Fukuoka Dental College, Fukuoka, Japan

**Keywords:** Nose and paranasal sinuses, Medication-associated necrosis of the jaws, Zoledronate, Purulent sinusitis

## Abstract

**Background:**

The management of bisphosphonate related necrosis of the jaw has become clinical routine. While approximately two thirds of the lesions are in the mandible, one third is located in the maxilla. In 40–50 % of maxillary necrosis the maxillary sinus is involved, leading to maxillary sinusitis and oro-antral communications.

**Methods:**

This retrospective single center study includes all patients with diagnosis of BP-ONJ of the maxilla and concomitant maxillary sinusitis. The information collected includes age, gender, primary disease, bisphosphonate intake, involving type of bisphosphonate, route of administration and duration of BP treatment previous to surgical treatment and treatment outcome.

**Results:**

A total of 12 patients fulfill the criteria of the diagnosis of maxillary sinusitis associated to maxillary necrosis, of which 6 Patients showed purulent sinusitis. All patients underwent surgical treatment with complete resection of the affected bone and a multilayer wound closure. A recurrence appeared in one patient with open bone and no sign of sinusitis and was treated conservatively.

**Conclusions:**

Purulent maxillary Sinusitis is a common complication of bisphosphonate-related necrosis of the maxilla. The surgical technique described can be suggested for the treatment of these patients.

## Background

Since its first description in 2003, reports of bisphosphonate related osteonecrosis of the jaw (BP-ONJ) accumulate. With the ability to reduce bone turnover through selective inhibition of osteoclasts, Bisphosphonates are used widespread in treatment of osteoporosis and bony metastases of malignant diseases. They are administered orally or intravenously, whereat the bioavailability of oral bisphosphonates is below 1 % [1]. Once circulating in the blood, 70 % are covalently bound to hydroxyapatite in bony tissues, the remainder is secreted via the kidneys. BPs bound to the bone are biologically inert, however, when absorbed by osteoclasts they lead to concentration dependent apoptosis via inhibition of Farnesyl-Pyrophosphate-synthase [2]. Being integrated only during bone turnover, concentration is suspected to be higher in areas of high turnover such as the alveolar processes [3]. Due to local factors like chewing forces, oral bacteria, the periodontal gap and a thin mucosa, the alveolar bone necessitates an elevated osteoclast-dependent bone turnover to maintain integrity [4]. When osteoclasts are diminished by a high local concentration of BPs, the bone is not capable to react to these local factors what may end in necrosis [5]. The prominent role of osteoclast inhibition in the pathogenesis of BP-ONJ is underlined by recent reports of osteonecrosis of the jaw following the treatment with Denosumab, a selective antibody against RANK-L and thus potent inhibitor of osteoclasts and its precursors, which have a similar incidence like BP-ONJ after the treatment with Zoledronate (ZOL), the BP with the highest antiresorptive potency [6].

The incidence of BP-ONJ is dependent on bisphosphonate type, route of administration and cumulative dose, underlying disease, gender, co-medication and oral health. It is lowest for oral treatment of primary osteoporosis (0.05-0.2 %) and highest for intravenous treatment of malignant diseases with bone metastases, intravenous administration of ZOL and additional treatment with inhibitors of angiogenesis or tyrosine-kinase (up to 20.5 %) [7].

Treatment suggestions of BP-ONJ differ. In the 2014 update on Medication related osteonecrosis of the jaws the American Association of Oral and Maxillofacial Surgeons (AAOMS) recommends surgical debridement or resection only in stage 2 and 3. Their approach has the major treatment goals to enable continued oncological therapy and preserve quality of life [8]. However, the favored treatment with antibacterial mouth rinse and antibiotic therapy only leads to freedom of symptoms in 53 % of the patients [9]. After promising results of a surgical approach, that can lead to a closed oral mucosa and absence of inflammation signs in 80-100 % of the cases, other national associations favor a complete necrosectomy with primary wound closure when the patients general condition allows it [10].

Roughly two thirds of the lesions occur in the mandible, only one third arises in the maxilla. While a plethora of articles present different perspectives of BP-ONJ, only few studies explicitly highlight the manifestation in the maxilla and only a case series of three patients exists for a defined treatment regime [11–15].

The aim of this study was to review our cases with maxillary BP-ONJ and concomitant sinusitis and to introduce a technique for their management.

## Method

This retrospective study includes all the patients with the diagnosis of bisphosphonates-related osteonecrosis of the maxilla and maxillary sinusitis that were operated in our department between 2007 and 2011. Patients without maxillary sinusitis, without a diagnosis consistent with BP-ONJ or with history of radiation therapy to the jaws were excluded. Data was collected using the hospital information system.

**Fig. 1:**
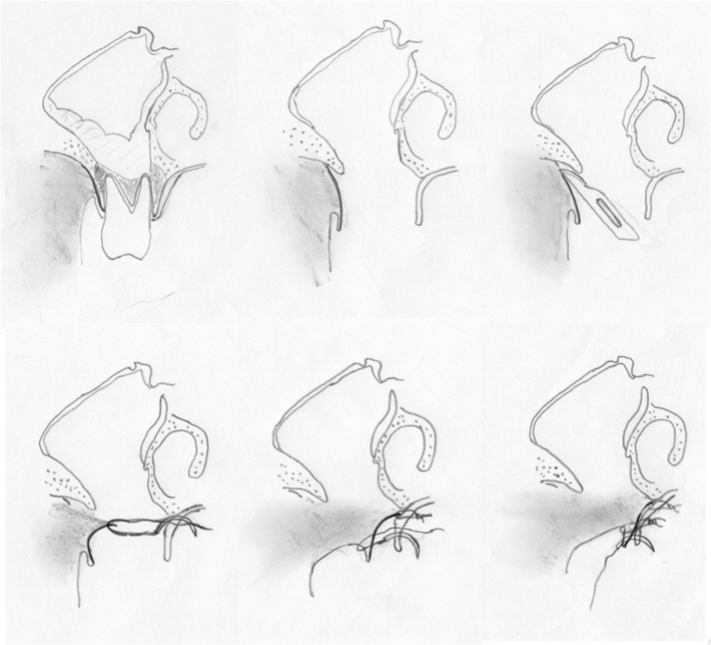
Schematic drawing of the technique

**Fig. 2:**
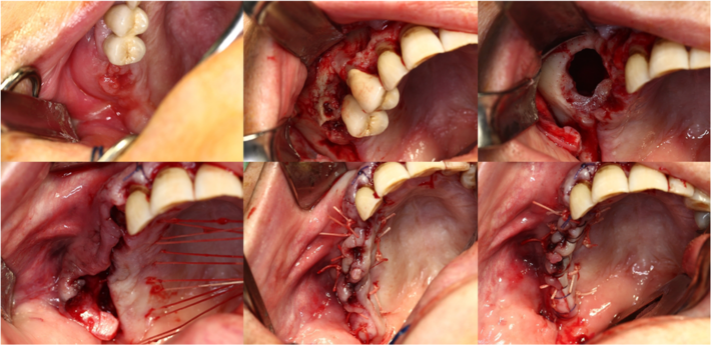
Clinical example of the necrosotomy and wound closure

All patients underwent surgery in general anesthesia after two days of preoperative intravenous antibiotic therapy with penicillin and metronidazole. Microbiological samples are taken at the time of initial contact with the patient and at the time of surgery when purulent drainage was visible. After elevating a mucoperiosteal flap using a crestal incision including areas of exposed bone, all the affected bone was removed with Luehr forceps and round burrs. Teeth in contact with the affected bone were removed, sharp bony edges rounded. After removal of the necrotic bone and thus opening of the maxillary sinus, polypoid mucosa was removed and the sinus was rinsed with iodine solution and xylometazoline. The sinus was endoscopically inspected. If the natural osteum was obstructed, it was widened with Weil’s forceps. The wound was closed using a multilayer technique previously described for the mandible and adapted for the maxilla [16]. In this technique, after slitting of the vestibular periosteum, its mobile part is quilted under the palatinal mucosa with absorbable backstitches. A second layer of absorbable backstitches is used to align the wound edges to one level and tighten the closure. A running suture brings the mucosal edges together and closes the wound (Figs. 1 and 2).

Postoperatively, patients were fed via a nasogastric feeding tube for five days. Intravenous antibiotic treatment was continued until discharging the patient after 6 days. Until removal of sutures 18–21 days postoperatively, patients were asked to only eat soft food, avoid blowing their noses and use xylometazoline nasal spray and inhalation with natural brine and chlorhexidine mouthwash frequently.

Postoperative controls were carried out for removal of sutures and then every six months. When recall in our unit was not feasible (e.g. distant place of residence) controls were carried out by the local dentist.

## Results

Twelve Patients met the inclusion criteria (10 female, 2 male) with a mean age of 67 years (range 55–82). Mean follow up time was 25 months (range 19 – 58). Mean duration of BP-treatment was 71 months (range 24–144 months), five patients received Zoledronate (mean 50 months, range 36–70), three patients were treated with Pamidronate (mean 117 months, 66–144 months), two with Ibandronate (mean 72 months, 48 and 96) and one with Alendronate (24 months) and Clodronate (84 months) each. Except for Alendronate and Clodronate, the route of administration was intravenously. The underlying diseases were breast cancer and multiple myeloma in five patients, and lung cancer and osteoporosis in one case respectively. BP-ONJ arose after tooth extraction in 6 patients. In 3 patients periodontitis was noted as initiating factor, which could not be clearly defined in the remaining three patients.

**Fig. 3:**
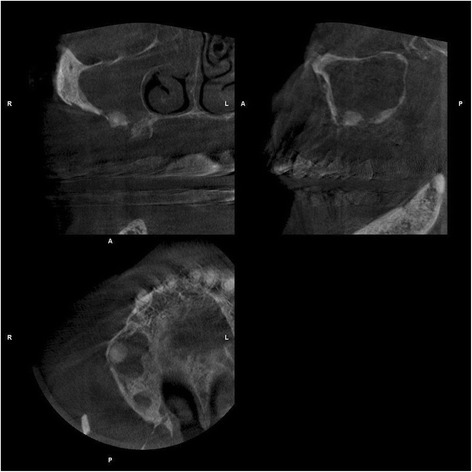
Cone beam CT showing a sequestrum and sinusitis in a patient without intraoral bone exposure

All patients were clinically classified BP-ONJ stage 3 [8]. Nine patients showed open bone lesions in the region of the second premolar or the first molar of one side of the maxilla, in two patients the incisor region was also involved. One Patient had intact mucosa of the toothless maxilla and no open bone intraorally but a sequestrum at the floor of the sinus with a purulent sinusitis (Fig. 3). In total, in six of the twelve patients the maxillary sinus was filled with ichor. Regarding the size of the defect three extensive defects with complete opening of the basal alveolar crest, seven large defects ranging from 15–30 mm and two small defects ranging von 5 to 15 mm were seen.

Eleven of twelve patients had an uneventful wound healing. One patient had a relapse of open bone 18 months after the surgery. However, the sinus was obstructed with a thick soft tissue scar. It was decided only to remove bony edges and sequestra not to endanger the delineation of the sinus. Two years later the patient showed up with an abscess of the ipsilateral cheek that was incised. Short time later she succumbed to the metastasizing breast cancer.

**Fig. 4:**
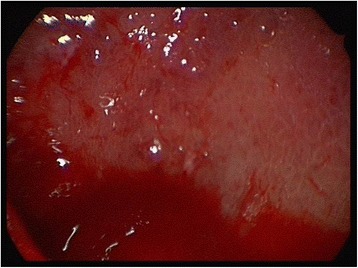
Remarkable whitish lines in the endoscopic view of an involved maxillary sinus

**Fig. 5:**
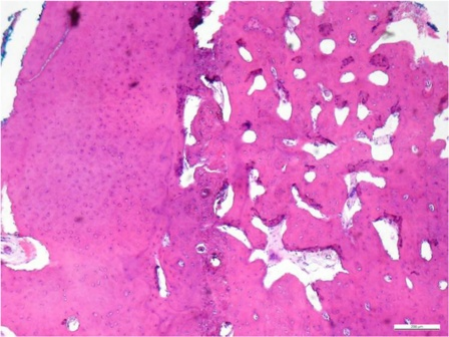
Histology of an antral wall showing regionally circumscribed necrotic and partially demineralized bone under a broadened mucosa

In all cases it was noticeable, that the Schneiderian membrane was easy to detach from the underlying bone. Endoscopic examination revealed whitish reticular lines of the sinus mucosa consistently (Fig. 4). Histology of the mucosa and the underlying bone was taken in selected cases and resulted in regionally circumscribed necrotic and partially demineralized bone under a broadened mucosa (Fig. 5). The antibiotic testing of the microbiological samples revealed no resistances against the antibiotic regimen used.

## Discussion

BP-ONJ is a side effect of antiresorptive treatment with growing importance that arises more frequently in the mandible than in the maxilla. The difference in bone architecture with its reduced blood supply might be an explanation, on the other hand, saliva and food particles accumulate in the mandible and might support local infections. Because of the good vascularization of the maxilla, osteomyelitis is rare in contrast to the mandible, where the thick cortical bone is nutritioned as an endartery system. Due to expansion of the maxillary sinus, the bony volume of the dorsal maxillary alveolar crest is mostly small, just reaching the root apices. Infections of the maxillary bone are likely to affect the maxillary sinus. The maxillary sinus itself is lined with a thin mucosa that early reacts to dental infections [17]. When periodontal infections or dental extractions lead to BP-ONJ of the dorsal maxilla, an involvement of the maxillary sinus is not unlikely: 40–50 % of the patients with a maxillary BP-ONJ show an ipsilateral maxillary sinusitis [12] and oro-antral fistulas occur in one third of the cases with maxillary BP-ONJ, and their management is demanding [15].

Triggering factors for BP-ONJ are tooth extractions and other dental surgical procedures, sharp bony edges and pressure marks. Other regions than the jaws are extremely rarely involved in Bisphosphonate-associated osteonecrosis. Some authors mention cutaneous manifestation such as dental sinus tracts [18], and only few case reports with Bisphosphonate-associated osteonecrosis of the external auditory canal (BPECO) are available [19]. The similarity of thin soft tissue coverage of the bone may explain a pathogenesis by minor self-inflicted trauma by regular aural toilet with cotton buds or fingers in the outer ear. Also secondary osteomyelitis can arise after otitis, mastoiditis or sinusitis and may lead to osteonecrosis.

Remarkably, the sinus mucosa in all patients of the present study was altered. Similarly to the oral mucoperiosteum, which can be very easily detached from the bone in patients treated with BPs, it could be removed from the underlying sinus bone without significant force. This supports the theory that soft tissue adjacent to bone is more affected by BPs than soft tissue that is not in connection with bone [20]. It is discussed that BPs are released from the bone by osteoclasts and low pH-values and then internalized by fibroblasts and epithelial cells [21]. Zoledronate inhibits proliferation and elevates apoptosis of these soft tissue cells, and inhibits expression of type-1-collagen [22]. As the periosteal membrane is connected to the bony surface by collagenous fibers, and type-1-collagen expression needs to be increased in maxillary mucosa healing, inhibition of fibroblasts and epithelial cells might explain the easier detachment of the periost and Schneiderian membrane [23]. Because vascular remodeling and neovascular formation is delayed, soft tissue regeneration may also be impaired in patients with BP-ONJ [24].

Treatment suggestions of manifest BP-ONJ differ. Whereas the first reports on management of BP-ONJ preferred a conservative approach, recent publications favor a surgical approach. While many authors describe treatment results between 84 and 100 % of mucosal healing after complete necrosectomy and thorough wound closure [16, 25, 26] healing of the necrosis occurs in only 25 % after strictly conservative measures and 28–58 % after partial resections or debridement without soft tissue closure [27, 28]. The more advanced regimes also reflect the argument that in most patients a continued treatment with BPs is necessary. This leads to higher concentrations in the jawbones resulting in even worse healing of bone and soft tissue wounds when surgery is done at a subsequent date. However, in its 2014 update of the position paper the AAOMS still recommends management with antibacterial mouth rinses and antibiotics in most patients [8]. In the experience of our group the conservative treatment of maxillary sinusitis did not lead to healing but to purulent discharge [13]. Persisting oro-antral communications can be covered with obturators, albeit ill-fitting dentures my lead to new osteonecrosis [11].

The technique proposed is adapted from a report of our group published in 2012 where five of the 21 lesions implicated the maxilla [16]. One of the patients in this current paper with multiple myeloma and Zoledronate treatment was already reported in the previous paper. In contrast to the technique described by Gallego et al. we tried not to use the pedicled buccal fat pad in the first surgical intervention in order to have more options in a possible later operation [14]. While the buccal fat pad provides a mechanic protection and a rich vascularization in the BRONJ site, in the technique used, the periosteum that is quilted under the palatal mucosa serves as a second layer for a reliable wound closure and helps to develop a thick scar that seals off the bone from the oral flora.

Interestingly, one of our patients who had a purulent sinusitis with a 9 × 8 mm measuring sequestrum did not show any exposed bone intra- or extraorally, and tooth extractions in the right maxilla had been carried out more than one year before. In the last years, the non-exposed variant has led to a discussion concerning the staging of BP-ONJ [29]. In most reports however, sites with the absence of a clear mucosal breakdown were declared as non-exposed BP-ONJ even though sinus tracts or periodontitis lesions existed. To our best knowledge a purulent maxillary sinusitis caused by a BP-ONJ sequestrum with completely bland oral mucosa has not been described before. As they may lead to pansinusitis, and orbital or intracranial implication, purulent sinusitis has the potential of life threatening complications and need to be treated early [30]. In our case low dose cone beam CT helped to assess the sequestrum and a complete unilateral opacification of the sinus, and the patient had no remaining symptoms after sequester removal, sinus surgery, and meticulous wound closure.

## Conclusions

Maxillary sinusitis and oro-antral communication associated to maxillary osteonecrosis is a severe complication of BP therapy. Cone beam computer tomography and endoscopy are helpful diagnostic and intraoperative tools. In combination with antibiotic treatment, the technique described can be suggested for the management of BP-ONJ and concomitant maxillary sinusitis.
